# Potential Roles of the Retinoblastoma Protein in Regulating Genome Editing

**DOI:** 10.3389/fcell.2018.00081

**Published:** 2018-07-31

**Authors:** Yuning Jiang, Wai Kit Chu

**Affiliations:** Department of Ophthalmology and Visual Sciences, The Chinese University of Hong Kong, Sha Tin, Hong Kong

**Keywords:** retinoblastoma, genome editing, CRISPR/Cas9, homologous recombination, Non-Homologous End-Joining

## Abstract

Genome editing is an important tool for modifying genomic DNA through introducing DNA insertion or deletion at specific locations of a genome. Recently CRISPR/Cas9 has been widely employed to improve the efficiency of genome editing. The Cas9 nuclease creates site-specific double strand breaks (DSBs) at targeted loci in the genome. Subsequently, the DSBs are repaired by two pathways: Homologous Recombination (HR) and Non-Homologous End-Joining (NHEJ). HR has been considered as “error-free” because it repairs DSBs by copying DNA sequences from a homologous DNA template, while NHEJ is “error-prone” as there are base deletions or insertions at the breakage site. Recently, *RB1*, a gene that is commonly mutated in retinoblastoma, has been reported to affect the repair efficiencies of HR and NHEJ. This review focuses on the roles of *RB1* in repairing DNA DSBs, which have impacts on the precision and consequences of the genome editing, both at the targeted loci and the overall genome.

## CRISPR/CAS9 and DNA double strand breaks

Gene editing technology is an important tool for editing genomic DNA through introducing DNA insertion or deletion at specific locations of the genome. Recently, CRISPR/Cas9 has been a popular technology for genome editing, which has potentials to be developed as a novel treatment for genetic diseases in human. Guided by RNA, which comprises a direct sequence of 20 nucleotides, the Cas9 endonuclease creates site-specific double strand breaks (DSBs) at targeted loci in the genome. Subsequently, it can knock out the targeted gene. Before the invention of CRISPR/Cas9 system, modifying the DNA sequence can be achieved by other methods, including site-directed mutagenesis and recombination based methods (Capecchi, 1989; Ling and Robinson, 1997). Site-directed mutagenesis is most useful in organisms such as bacteria and yeast, with relatively simple laboratory methods for the introduction and selection of a desired mutation (Storici et al., 2001). One established protocol utilizes polymerase chain reaction (PCR)-mediated methods and oligonucleotides containing the desired mutation to amplify the template DNA. The newly synthesized DNA would then carry the desired mutation. The recombination based method employs cell transformation with exogenous DNA carrying DNA sequence homologous to the endogenous DNA. If DSBs are created in the endogenous DNA, the homologous recombination machinery would repair the DSBs by copying the exogenous DNA. By using this method, insertion or deletion of the target DNA sequence could be introduced into the desired loci. This recombination based method is commonly used in mammalian cells (Capecchi, 1989). Importantly, this recombination based method relies on the occurrence of DSBs at the desired locus. DSBs could be generated endogenously or exogenously (Hartlerode and Scully, 2009). Endogenous DSBs could arise from DNA replication fork encountering a broken template or from specific physiological activities such as meiosis or V(D)J recombination at the immunoglobulin heavy chain locus. Exogenously, DSBs could be generated by exposing cells to DNA damaging agents such as ionizing radiation, UV light or topoisomerase poisons. As a result, the occurrence rate of DSBs at the desired locus is varying. At some loci, the chance of having a DSB could be extremely low, resulting in a very low genome-editing efficiency at these loci. In addition, the appearance of DSBs in other undesired loci may lead to erroneous recombination and “off-target” editing at those loci. Therefore, the CRISPR/Cas9 technology could solve both the low DSB generation and the “off-target” issues by inducing DSBs at specific loci.

DSBs are thought to be the most cytotoxic DNA damage compared to other types of DNA damages, such as DNA mismatches and base damages, because of the discontinuation of both DNA strands. It has been reported that the DSBs induced by Cas9 are toxic to human pluripotent stem cells, which would limit the efficiency of genome editing (Ihry et al., 2018). This toxic response is dependent on p53 (Haapaniemi et al., 2018). These results suggest that p53 inhibition may improve the efficiency of genome editing. And importantly, the p53 status should be closely monitored in cells that have been treated with CRISPR–Cas9.

There are two main pathways to repair DSBs: Homologous Recombination (HR) and Non-Homologous End-Joining (NHEJ). HR has been considered as “error-free” because it repairs DSBs by copying DNA sequences from a homologous DNA template, while NHEJ is “error-prone” because it can lead to small deletions or insertions at the breakage site. The choice of repairing DSBs through HR or NHEJ would affect the genetic outcome of the CRISPR/Cas9 genome editing. For example, if no exogenous DNA is provided, repairing DSBs by NHEJ is desirable to generate deletions or insertions at the specific loci. And usually it is difficult to control the length of these NHEJ mediated deletions and insertions. If HR is used to repair DSBs when there is no exogenous template, the DNA breakage would be repaired by copying DNA sequence from the sister chromatid or the homologous allele, which have identical or highly similar DNA sequence. Therefore the outcome would be no or limited mutation at the designated loci. On the other hand, if an exogenous template carrying a designated mutation is provided, HR would be desirable to create precise DNA sequence deletion, insertion or replacement. Therefore, it is important to understand how cells choose between HR and NHEJ to repair DSBs generated by CRISPR/Cas9.

## Mechanisms of DNA DSB repair

**Figure 1:**
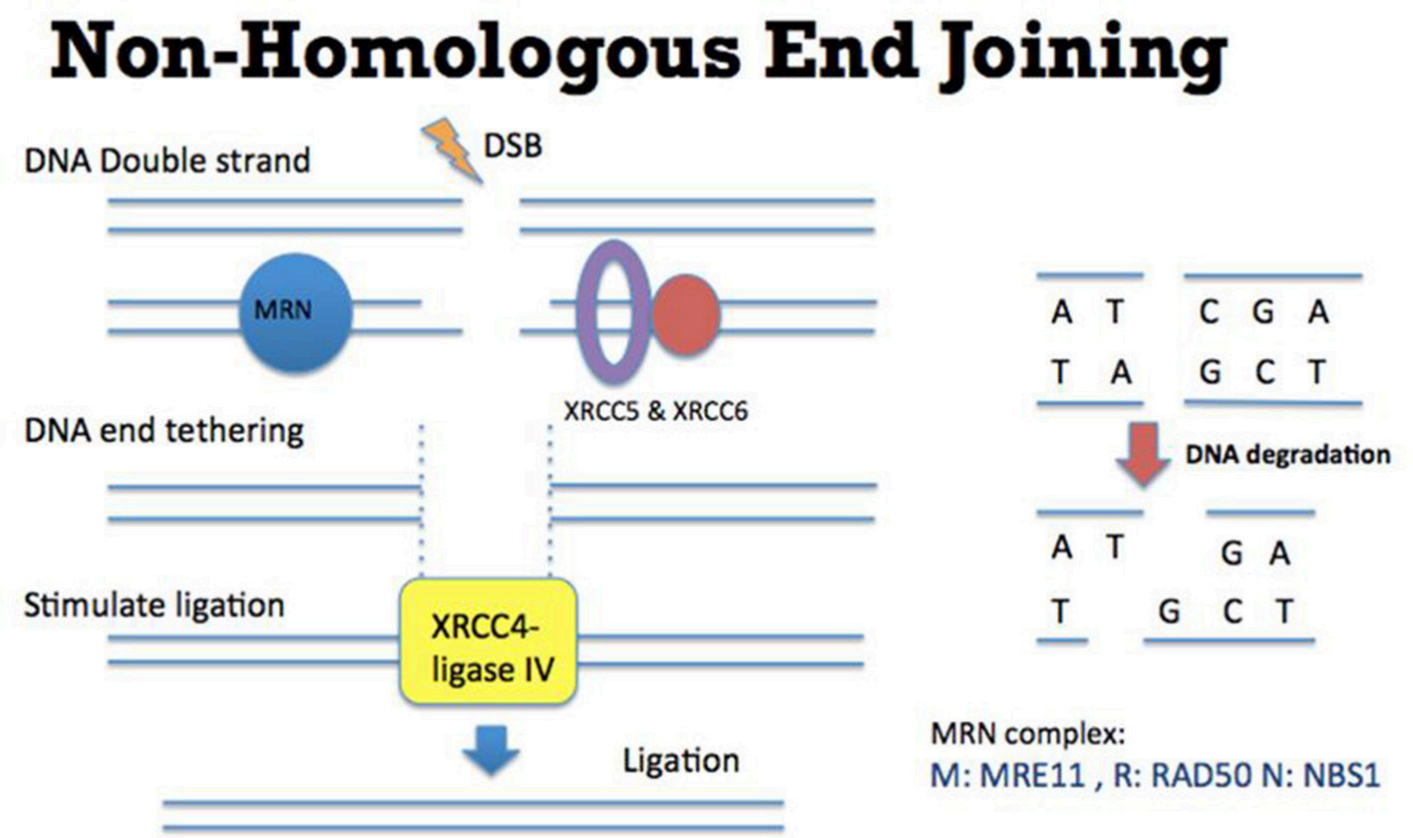
Mechanism of Non-Homologous End Joining (NHEJ). **(Left)** DSB ends are tethered by MRN complex (comprised of MRE11, RAD50, and NBS1) (blue), XRCC5, and XRCC6 (purple and red). XRCC4-ligase IV (yellow) is recruited to DSB ends to stimulate the DNA ligation. **(Right)** In this NHEJ mediated DSB repair pathway, some DNA sequences might be degraded at the DNA breakage site. Therefore this repair mechanism could potentially generate some “errors” (such as losing nucleotides as indicated by C and A) in repairing DSBs.

**Figure 2:**
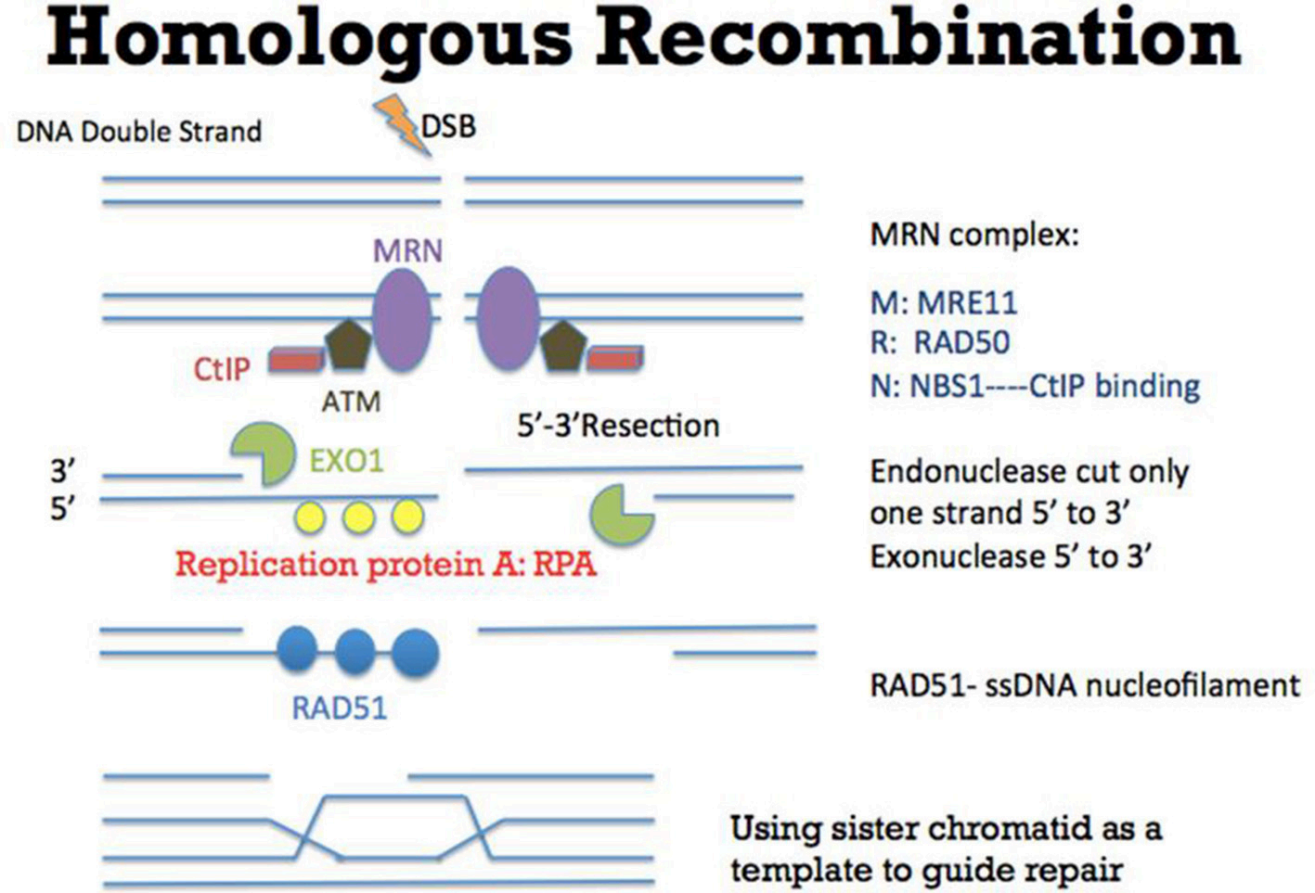
Mechanism of Homologous Recombination (HR). As an error-free pathway of DSB repair, multiple proteins such as CtIP (red), ATM (brown), and MRN complex (comprised of MRE11, RAD50, and NBS1) (purple) are recruited to initiate DSB repair. At the DSB ends, EXO1 (green) resects one strand of DNA directionally (5′-3′) to generate a 3′ single strand DNA (ssDNA) tail. Replication protein A (RPA) (yellow) then binds to the ssDNA tail, which is subsequently replaced by another protein RAD51 (blue). During this step, other proteins such as BRCA2 (not shown in this figure) would help RAD51 to bind to ssDNA to form the RAD51-ssDNA nucleofilament (Esashi et al., 2007; Ayoub et al., 2009). The nucleofilament could then search for the homologous DNA sequences, invade into the homologous DNA sequences and synthesize DNA by using the homologous DNA sequences as templates. After the DNA synthesis, the DNA ends are ligated to the other side of the DSB.

Faithful repair of damaged DNA is important for genome integrity. Many types of DNA lesions lead to DNA DSB formation with loss of continuity of genome (Aparicio et al., 2014). Unfaithful repair of DSBs can lead to serious consequences, including cancer promoting initiation, disease progression and therapy resistance. There are two main pathways to repair DSBs: NHEJ and Homologous Recombination (HR). Theoretically NHEJ could take place throughout the entire cell cycle although some studies suggested that NHEJ has dominant roles in the G0 and G1 stages (Karanam et al., 2012; Chiruvella et al., 2013). In this DNA repair mechanism (Figure 1), many factors such as XRCC5 (also known as Ku80), XRCC6 (also known as Ku70), DNA-PKcs, XRCC4-ligase IV, and MRE11-RAD50-NBS1 (MRN) complex are required for direct ligation of DNA ends (Lieber et al., 2003). It is worth to be noted that some DNA sequences might be degraded at the DNA breakage sites. Therefore, this repair mechanism could potentially generate some “errors” in repairing DSBs. Nevertheless, NHEJ is a relatively fast and simple mechanism, compared to HR, to repair DSBs (Difilippantonio et al., 2000). In addition, NHEJ is also critical for the generation of diversity at the immunoglobulin gene (Critchlow and Jackson, 1998). Apart from NHEJ, HR is the other DSB repair pathway (Figure 2). HR takes place predominately in the S and G2 phases of the cell cycle (Kato et al., 1993). In these two cell cycle stages, DNA is being replicated or has been replicated. Therefore the sister chromatids, which carry identical DNA sequences, are available to serve as templates for HR. As an error-free pathway of DSB repair, a significant difference from NHEJ is the directional (5′-3′) degradation of DSBs to generate a 3′ single strand DNA (ssDNA) tail. Recent studies have identified multiple proteins in this DNA resection reaction, including BLM, DNA2, EXO1, CtIP, and MRN complex (comprised of MRE11, RAD50, and NBS1; Nimonkar et al., 2011). Replication protein A (RPA) then binds to the ssDNA tail, which is subsequently replaced by another protein RAD51. During this step, other proteins such as BRCA2 and RAD54 would help RAD51 to bind to ssDNA to form the RAD51-ssDNA nucleofilament (Esashi et al., 2007; Ayoub et al., 2009). The nucleofilament could then search for the homologous DNA sequences, invade into the homologous DNA sequences and synthesize DNA by using the homologous DNA sequences as templates. After the DNA synthesis, the DNA ends are then ligated to the other side of the DSBs.

It has been reported that NHEJ and HR are in competition for the repairing of DSBs: when NHEJ is upregulated, HR is then inhibited and vice versa. And the DNA resection step has been proposed to be one of the major determinants to regulate the NHEJ and HR efficiencies (Deriano and Roth, 2013; Jasin and Rothstein, 2013). If there is no DNA resection, the DSB ends could be directly ligated and be repaired through the NHEJ pathway. When there is extensive DNA resection, the ssDNA tail could invade homologous sequences to repair DSBs through the HR pathway.

## RB and the multiple functions in genome stability

Biallelic inactivation of *RB1* gene is a disease causing mutation of retinoblastoma (Rushlow et al., 2013), the most common intraocular tumor of childhood with an incidence rate of 1 in 14,000–18,000 live births. It accounts for 3% of the cancers occurring in children younger than 15 years of age (Rodriguez-Galindo et al., 2003). Retinoblastoma has been reported to arise from the cone precursor cells in retina (Xu et al., 2009). Late stage of retinoblastoma could invade into the sclera and the orbit, or even to the systemic extra-central nervous system. Retinoblastoma occurs in both heritable (25%) and non-heritable (75%) forms. Loss of function of *RB1* gene in sporadic retinoblastoma could result from the mutations of both *RB1* alleles, or with the loss of heterozygosity (LOH; Choy et al., 2002). In human embryonic stem cells, knocking out *RB1* gene by CRISPR/Cas9 would lead to the formation of neural enriched teratomas, which mimic the trilateral retinoblastoma tumors (Avior et al., 2017). Similarly, knocking out of *Rb1* and *Retinoblastoma-like 1* (*Rbl1*) genes by CRISPR/Cas9 led to retinoblastoma formation in *Xenopus tropicalis* (Naert et al., 2016; Naert and Vleminckx, 2018). *RB1* gene codes for the RB protein, which is a multifunctional protein participating in several various molecular processes. The classical function of RB is well known for its ability to repress transcription, which in turn regulate cell cycle progression and cell proliferation (Manning and Dyson, 2012). In G1 phase, RB protein could be phosphorylated by cyclin-dependent kinases (CDKs) 4 and 6. The phosphorylated RB could not bind to a transcription factor E2F (Ewen et al., 1993; Kato et al., 1993). Consequently, the RB-unbound E2F binds to the promotors and induces the expressions of several target genes, which allow cells to progress into S phase. Also, it has been reported that throughout the cell cycle, RB is sumoylated at early G1 phase (Meng et al., 2016). SUMOylation of RB could stimulate its phosphorylation and is required for cell proliferation. In addition, deregulation of microRNAs (miRNAs) has also been reported to involve in various stages of retinoblastoma. These miRNAs could be used as diagnostic, prognostic and therapeutic biomarkers in retinoblastoma patients (Golabchi et al., 2018). For example, miR-622 was able to inhibit the expression of RB by directly targeting its 3′ untranslated region (Ma et al., 2015). Furthermore, miR-503-5p overexpression could also downregulate the expression of the RB/E2F signaling pathway proteins (Li et al., 2017). Besides this classical cell cycle controlling the function, RB protein has been reported to possess functions in suppressing chromosome instability (CIN) and aneuploidy (Coschi and Dick, 2012; Manning and Dyson, 2012). In RB knocked down cells, chromosomes were found missegregated frequently with defective centromere and telomere maintenance. In addition, mouse Rb was found to control chromatin cohesion by the interaction between the LXCXE amino acid domain of Rb and the cohesion II subunit Cap-D3 (Isaac et al., 2006; Coschi et al., 2010). These results suggest roles of RB inactivation and genome instability, which could be one of the mechanisms in tumorigenesis.

**Figure 3:**
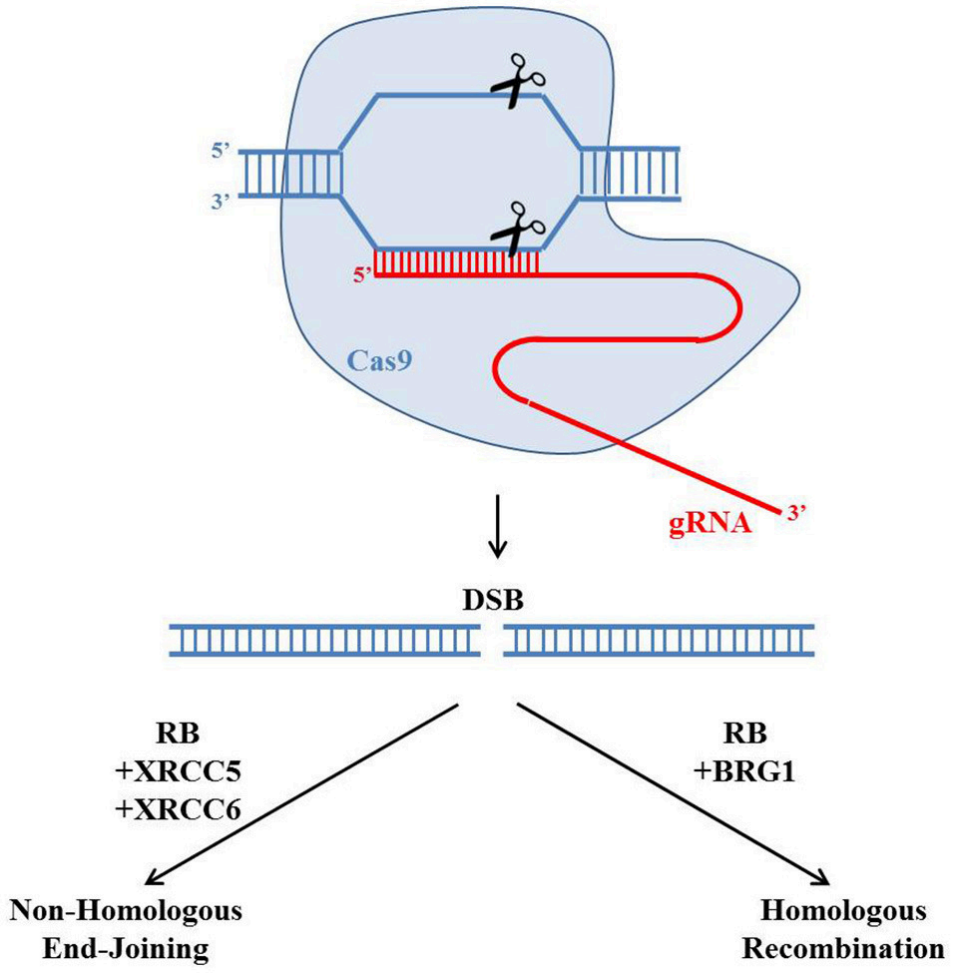
Potential roles of the retinoblastoma protein in regulating CRISPR/Cas9 genome editing. The gRNA (red) could guide the Cas9 endonuclease (blue) to create a site-specific double strand break (DSB). There are two main pathways to repair the DSB: Homologous Recombination (HR) and Non-Homologous End-Joining (NHEJ). Recently RB has been reported to regulate both HR and NHEJ by forming protein complexes with BRG1 or XRCC5 and XRCC6, respectively. The choice of repairing DSBs through HR or NHEJ would affect the genetic outcome of the CRISPR/Cas9 genome editing.

In particular, in human cells, RB inactivation led to accumulation of DSBs (Pickering and Kowalik, 2006). Depletion of RB in U2OS, a human osteosarcoma cell line, showed higher numbers of γH2AX foci, which represent the locations of DSBs (Rogakou et al., 1998). And the number of these γH2AX foci could be further elevated by treating DSB inducing ionizing radiation. Importantly, these γH2AX foci persisted longer time in RB depleted cells. In both osteosarcoma and breast cancer cell lines, RB depletion led to lower cell survival rates in response to other DSB inducing drugs such as topoisomerase poisons etoposide and camptothecin (Velez-Cruz et al., 2016). These observations led to a hypothesis that RB may regulate the DSB repair pathways. Indeed, RB has been reported to regulate both HR and NHEJ recently (Figure 3). RB was found to recruit another protein BRG1 to DSBs to induce DNA resection to initiate HR (Velez-Cruz et al., 2016). BRG1 belongs to the SWI/SNF family of ATPases, which could remodel chromatin structure to undergo DNA resection. In BRG1 depleted cells, DNA resection was found to be reduced (Velez-Cruz et al., 2016). Importantly, the recruitment of RB and BRG1 proteins to DSBs required the ATM mediated phosphorylation of serine 29 on E2F1, a well-established protein partner of RB. Interestingly, another clinical observation found that in women with high-grade serous epithelial ovarian cancer, HR deficiency and *RB1* loss were correlated (Garsed et al., 2018). These results suggest that RB is important for HR.

In addition to HR, RB was also reported to play important roles in NHEJ (Cook et al., 2015). In RB knocked-down cells, the NHEJ efficiency was reduced while the radiation induced chromosomal aberration was elevated, probably through the protein interactions between RB and core members of the NHEJ pathway such as XRCC5 and XRCC6. This study suggests that RB is important for NHEJ.

CRISPR/Cas9 technology is a powerful technology to generate locus specific DSBs. Despite, it is worth noted that there are still potential off-target events generated by CRISPR/Cas9. On the other hand, improving the genome editing efficiency may comprise the accuracy. The balance of genome editing efficiency and accuracy could be greatly affected by regulating the efficiencies of HR and NHEJ. Recent studies have identified functional roles of RB in both HR and NHEJ, implying RB could be a key determinant of the CRISPR/Cas9 mediated genome editing. The choice of repairing DSBs through HR or NHEJ would affect the genetic outcome of the CRISPR/Cas9 genome editing. As HR and NHEJ have been reported to compete with each other for the repairing of DSBs, it is important to investigate the relationships between RB, HR, and NHEJ (Deriano and Roth, 2013; Jasin and Rothstein, 2013). Furthermore, both RB, HR, and NHEJ exhibit cell cycle specific roles, which could affect the genetic outcome of CRISPR/Cas9 mediated genome editing at various cell cycle stages.

## Author contributions

All authors listed have made a substantial, direct and intellectual contribution to the work, and approved it for publication.

### Conflict of interest statement

The authors declare that the research was conducted in the absence of any commercial or financial relationships that could be construed as a potential conflict of interest.
